# Pathological Tau From Alzheimer’s Brain Induces Site-Specific Hyperphosphorylation and SDS- and Reducing Agent-Resistant Aggregation of Tau *in vivo*

**DOI:** 10.3389/fnagi.2019.00034

**Published:** 2019-03-05

**Authors:** Jin Miao, Ruirui Shi, Longfei Li, Feng Chen, Yan Zhou, Yunn Chyn Tung, Wen Hu, Cheng-Xin Gong, Khalid Iqbal, Fei Liu

**Affiliations:** ^1^Key Laboratory of Neuroregeneration of Jiangsu and Ministry of Education of China, Co-innovation Center of Neuroregeneration, Nantong University, Nantong, China; ^2^Department of Neurochemistry, Inge Grundke-Iqbal Research Floor, New York State Institute for Basic Research in Developmental Disabilities, Staten Island, NY, United States; ^3^Laboratory Animal Center, Nantong University, Nantong, China

**Keywords:** Alzheimer’s disease, AD P-tau, hyperphosphorylation of tau, tau pathology, propagation of tau pathology

## Abstract

Neurofibrillary tangles (NFTs) made up of hyperphosphorylated tau are a histopathological hallmark of Alzheimer’s disease (AD) and related tauopathies. Hyperphosphorylation of tau is responsible for its loss of normal physiological function, gain of toxicity and its aggregation to form NFTs. Injection of misfolded tau seeds into mouse brain induces tau aggregation, but the nature of tau phosphorylation in pathologic tau seeded pathology is unclear. In the present study, we injected hyperphosphorylated and oligomeric tau isolated from AD brain (AD P-tau) into hippocampus of human tau transgenic mice and found that in addition to tau aggregation/pathology, tau was hyperphosphorylated at Ser202/Thr205, Thr212, Ser214, Thr217, Ser262, and Ser422 in AD P-tau injected hippocampus and at Ser422 in the contralateral hippocampus and in the ipsilateral cortex. AD P-tau-induced AD-like high molecular weight aggregation of tau that was SDS- and reducing agent-resistant and site-specifically hyperphosphorylated in the ipsilateral hippocampus. There were no detectable alterations in levels of tau phosphatases or tau kinases in AD P-tau-injected brains. Furthermore, we found that hyperphosphorylated tau was easier to be captured by AD P-tau and that aggregated tau was more difficult to be dephosphorylated than the non-aggregated tau by protein phosphatase 2A (PP2A). Based on these findings, we speculate that AD P-tau seeds hyperphosphorylated tau to form aggregates, which resist to the dephosphorylation by PP2A, resulting in hyperphosphorylation and pathology of tau.

## Introduction

Alzheimer’s disease (AD) is multifactorial and involves different etiopathogenic mechanisms (Iqbal et al., 2005, 2016; Iqbal and Grundke-Iqbal, 2010). Histopathologically, AD is characterized by intraneuronal neurofibrillary tangles (NFTs) and extracellular deposits of β-amyloid plaques. Clinicopathological correlation studies have shown that the number of NFTs, but not of amyloid plaques, correlates with the degree of dementia in AD patients (Tomlinson et al., 1970; Alafuzoff et al., 1987; Arriagada et al., 1992; Quiroz et al., 2018).

NFTs initiate in subcortical regions, transentorhinal area, and entorhinal cortex, then appear in the hippocampal formation and some parts of the neocortex, followed by most of the neocortex—the Braak stages—whereas the distribution of NFTs correlates with the progression of the disease (Braak and Braak, 1991; Braak and Del Tredici, 2011). Tau pathology in AD develops progressively in regions of the brain with known synaptic connectivity. Recently, tau tracer retention measured by positron emission tomography also showed similar stages (Johnson et al., 2016; Schöll et al., 2016; Schwarz et al., 2016). Thus, the regional distribution of tau pathology is apparently associated with the disease progression.

NFTs are composed of hyperphosphorylated and aggregated microtubule-associated protein tau (Grundke-Iqbal et al., 1986a,b), major function of which is to promote microtubule assembly and maintain microtubule structure. This biological activity of tau is regulated by its degree of phosphorylation. In AD brain, tau is abnormally hyperphosphorylated. The hyperphosphorylation inhibits the activity of tau to promote microtubule assembly (Lindwall and Cole, 1984; Iqbal et al., 1986; Alonso et al., 1994). Unlike normal tau, the hyperphosphorylated and oligomeric tau isolated from AD brain (AD P-tau) sequesters/captures normal tau and templates it into filaments *in vitro* (Alonso et al., 1994). This phenomenon was recently termed prion-like property of pathological tau.

Injection of brain extract from tau_P301S_-expressing mice into the brain of transgenic wild-type tau-expressing mice induces tau aggregation not only at the injection sites, but also in the anatomically connected brain regions in a time-dependent manner, introducing the concept of “propagation of tau pathology” (Clavaguera et al., 2009). Subsequently, several studies reported the induction of tau pathology by intrahippocampal injection of misfolded tau seeds (Liu et al., 2012; de Calignon et al., 2012; Iba et al., 2013; Ahmed et al., 2014; Dujardin et al., 2014; Peeraer et al., 2015). We showed that injection of AD P-tau into the hippocampi of Tg/hTau and 3xTg-AD mice induces AD-like NFTs, which can be labeled by various phosphorylation-dependent and site-specific anti-tau antibodies (Hu et al., 2016; Dai et al., 2018). However, whether AD P-tau induces tau hyperphosphorylation is not documented and the possible mechanism(s) involved is unknown. In the present study, we analyzed tau phosphorylation in AD P-tau-injected hippocampus in Tg/hTau mice and found site-specific hyperphosphorylation and SDS- and reducing agent-resistant high molecular weight smears of tau, but no alteration in the levels of tau phosphatases or kinases in AD P-tau injected hippocampus. Thus, the AD P-tau-seeded tau aggregation/pathology apparently maintains its characteristics.

## Materials and Methods

### Animals

The hemizygous human tau transgenic [Tg/hTau, B6.Cg-Mapttm1 (EGFP)Klt Tg(MAPT) 8cPdav/J] mice with murine tau knockout (tau−/−) background (Duff et al., 2000) and Tau−/− mice (Tucker et al., 2001) were obtained from Jackson Laboratory (Bar Harbor, ME, USA) and generated by crossing Tg/hTau and Tau−/−. The mice were housed under a 12-h light/dark cycle, with access to food and water *ad libitum*. All animal handling and use were as per the protocol approved by Institutional Animal Care and Use Committee at New York State Institute for Basic Research in Developmental Disabilities in accordance with the PHS Policy on Human Care and Use of Laboratory Animals.

### Preparation of Hyperphosphorylated and Oligomeric Tau (AD P-tau), Heat-Stable Tau (HS-tau) and Sarkosyl Insoluble Tau (SI-tau) From AD Brain

Frozen brain tissue samples from autopsied and histopathologically confirmed AD cases were obtained from the Brain Tissue Resource Center, McLean Hospital, Belmont, MA, USA. The use of autopsied frozen human brain tissue was in accordance with the National Institutes of Health guidelines and was exempted by the Institutional Review Board (IRB) of New York State Institute for Basic Research in Developmental Disabilities because “the research does not involve intervention or interaction with the individuals” nor “is the information individually identifiable.”

Hyperphosphorylated and oligomeric tau (AD P-tau) was isolated from autopsied and frozen AD cerebral cortex as described by us previously (Köpke et al., 1993; Hu et al., 2016). Briefly, 10% brain homogenate prepared in the buffer (20 mM Tris-HCl, pH 8.0, 0.32 M sucrose, 10 mM β-mercaptoethanol, 5 mM MgSO_4_, 1 mM EDTA, 10 mM glycerophosphate, 1 mM Na_3_VO_4_, 50 mM NaF, 2.0 mM benzamidine, 1.0 mM 4-(2-aminoethyl) benzenesulfonyl fluoride hydrochloride (AEBSF), and 10 μg/ml each of aprotinin, leupeptin, and pepstatin) was centrifuged at 27,000× *g* for 30 min. The pellet was saved for sarkosyl insoluble tau (SI-tau) preparation. The supernatant was further centrifuged at 235,000× *g* for 45 min, and the resulting pellet, i.e., AD P-tau, was collected and washed three times and then resuspended in saline. The supernatant was used for heat stable tau (HS-tau) preparation.

HS-tau preparation: the supernatant from above 235,000× *g* was adjusted to 0.75 M NaCl and 10 mM β-mercaptoethanol, heated for 5 min at 100°C, and centrifuged at 25,000× *g* for 30 min. The supernatant was dialyzed against 10 mM Tris-HCl, pH 7.6, and concentrated by five times.

Sarkosyl insoluble aggregated tau (SI-tau) preparation: the pellet from above 27,000× *g* was homogenized in the homogenization buffer containing 0.1% sarkosyl and centrifuged at 10,000× *g* for 10 min. The supernatant was adjusted to 1% sarkosyl, incubated for 1 h at room temperature, and centrifuged at 235,000× *g* for 45 min. The pellet was collected as SI-tau after washing with 50 mM Tris-HCl for two times.

### Stereotaxic Injection

AD P-tau was injected into the right hippocampus in Tg/hTau mice as described previously (Hu et al., 2016; Dai et al., 2018). Briefly, mice were deeply anesthetized with 1.25% Avertin (Sigma, St. Louis, MO, USA) and placed on a stereotaxic frame. After craniotomy, 1 mm in diameter, was made with a motorized mini-drill, the tau seeds were injected using a 10 μl Hamilton syringe custom made with a 30 gauge/0.5 inch/hypodermic needle (Hamilton Syringe Co., Reno, NV, USA). AD P-tau was unilaterally injected into the right hippocampus (0.55 μg in 2.0 μl saline per hippocampus) in 9–11-month-old Tg/hTau or Tau−/−mice. The coordinates were as follows: −2.5 mm anterior/posterior, +2.0 mm medial/lateral to Bregma, and −1.67 mm dorsal/ventral to dura surface. AD P-tau was injected at a rate of 1.25 μl/min, and the needle was kept in position for three additional minutes before slow withdrawal to prevent leakage of the liquid infused. Saline was injected into Tg/hTau or Tau−/− mice of the same age as vehicle controls. The skin was sutured after injection, and the mice were allowed to completely recover on a soft warming pad before they were returned to their home cages.

### Immunohistochemistry

At 10 weeks after injection, mice were deeply anesthetized and transcardially perfused with saline followed by buffered 4% paraformaldehyde. The whole brain was collected, post fixed in the same fixative overnight at 4°C, and dehydrated in buffered 30% sucrose solution. The brains were then cut into 40-μm serial coronal sections using a freezing microtome, and the free-floating sections were preserved in antifreeze solution at −20°C until used for immunohistochemical staining. Sections were washed with PBS, permeabilized with 0.3% Triton X-100, blocked with normal goat serum, and then incubated with primary antibody overnight at 4°C. Then, the sections were incubated with Alexa Fluor 555- or 488- goat anti-mouse or rabbit IgG (1:1,000, Life Technologies, Rockford, IL, USA) or a combination where appropriate. Images were captured with an EZ-C1 laser scanning confocal microscope (Nikon Instruments, Melville, NY, USA) and the Z-stack function was used to reveal the morphology of tangles. Control staining with samples which are known to be positive/negative for target antigen and the control with the absence of primary antibody were included in each experiment. AT8 staining was thresholded using Yen’s arithmetic and quantified using the ImageJ software package. Area of AT8-positive somatodendritic profiles in the hippocampus was measured based on 3–4 sections each of Tg/hTau mice injected with AD P-tau.

### Western Blots

Ipsilateral and contralateral hippocampi and cortices were dissected and homogenized in cold buffer consisting of 50 mM Tris-HCl, pH 7.4, 2.0 mM EDTA, 2.0 mM EGTA, 10 mM β-mercaptoethanol, 150 mM NaCl, 1.0 mM Na_3_VO_4_, 50 mM NaF, 10 μg/ml aprotinin, leupeptin and pepstatin, and 0.5 mM AEBSF. The homogenates were boiled in 1× Laemmli buffer (125 mM Tris-HCl, pH 6.8, 2% SDS, 10% glycerol, 2.5% β-mercaptoethanol, 0.004% bromophenol blue) for 5 min. Protein concentration was quantified by using A660 Protein Assay kit (Pierce, Rockford, IL, USA). The same amount of brain homogenate proteins was separated by SDS-PAGE and electrically blotted onto polyvinylidene fluoride membrane (PVDF, Millipore). After blocking with 5% milk in Tris-HCl buffered saline (TBS), the membrane was incubated with primary antibodies (Table 1) and followed by the species-matched peroxidase-conjugated secondary antibodies (Jackson ImmunoResearch, West Grove, PA, USA). The blots were then developed by using ECL kit (Thermo Fisher Scientific) and exposed to HyBlot CLr autoradiography film (Denville Scientific, Inc., Holliston, MA, USA). Immunoblotting image was quantified by using the Multi Gauge software V3.0 from Fuji Film (Minato, Tokyo, Japan).

**Table 1:** Primary antibodies used in the present study.

Antibody	Type	Species	Specificity	Source/reference (catalog/lot #)
R134d	Poly-	R	Total tau	In-house (Tatebayashi et al., 1999)
Anti-pS199	Poly-	R	pSer199	Invitrogen (44734G)/0300A
Anti-pT205	Poly-	R	pThr205	Invitrogen (44–738G)/RJ239402
AT8	Mono-	M	pSer202/pThr205	Thermo Scientific (MN1020/PI205175)
Anti-pT212	Poly-	R	pThr212	Invitrogen (44740G)/1709582A
Anti-pS214	Poly-	R	pSer214	Invitrogen (44–742G)0500B
Anti-pT217	Poly-	R	pSer217	Invitrogen (44–744)/785771A
Anti-pS262	Poly-	R	pSer262	Invitrogen (44–750G)/QK220618
Anti-pS396	Poly-	R	pSer396	Invitrogen (44752G)/567847B
Anti-pS404	Poly-	R	pSer404	Invitrogen (44–758G)/5G255476
R145d	Poly-	R	pSer422	In-house (Pei et al., 1999)
PHF-1	Mono-	M	pSer396/pSer404	Dr. Peter Davies
Tau-1	Mono-	M	Up-tau (195–202)	Dr. Binder, L. I.
Anti-pERK	Poly-	R	pThr202/pTyr204	Cell Signaling (4377S/10)
Anti-pJNK	Poly-	R	pThr183/pTyr185	Cell Signaling (9251S/10)
Anti-pAKT	Poly-	R	pSer473	Cell Signaling (4058L/30)
Anti-pGSK-3β	Mono-	R	pSer9	Cell Signaling (9323S/13)
Anti-pAMPK	Mono-	R	pThr172	Cell Signaling (2535L/16)
Anti-pP70S6K	Mono-	R	pThr389	Cell Signaling (9234S/11)
Anti-PKAcα	Poly-	R	Total PKAc	Santa Cruz (SC-9031/B1111)
Anti-Cdk5	Mono-	M	Total Cdk5	Santa Cruz (SC-249/G1817)
8D9	Mono-	M	Total Dyrk1A	In-house (Wegiel et al., 2004)
CK1ε	Mono-	M	Total CK1ε	Santa Cruz (SC-81446)
Anti-PP2Ac	Mono-	M	Total PP2Ac	BD Transduction (610556/26637)
Anti-DM-PP2Ac	Mono-	M	Demethylated Lys309	Santa Cruz (SC-80990/F2512)
R126	Poly-	R	Total PP2B	In-house (Pei et al., 1998)
Anti-PP5	Poly-	R	Total PP5	Bahl et al. (2001)
PP1	Poly-	R	Total PP1	Santa-Cruz (sc-7482/1032)
Anti-GAPDH	Poly-	R	GAPDH	Sigma (G9545/015M4824V)
Anti-β-actin	Mono-	M	β-actin	Sigma (A1978/046M4789V)

*Abbreviations: Mono-, monoclonal; p-, phosphorylated; up-, unphosphorylated; Poly-, polyclonal; M, Mouse; R, Rabbit*.

### Dephosphorylation by Protein Phosphatase 2A (PP2A)

Heat stable tau (HS-tau) and sarkosyl insoluble tau (SI-tau) were dephosphorylated with protein phosphatase 2A (PP2A) for various time points in the buffer (100 mM Tris-HCl, pH7.4, 1 mM MnCl_2_, 10 mM β-mercaptoethanol). The dephosphorylation products were then analyzed for phosphorylation by dot-blots developed with anti-pS199-tau (Table 1).

### Tau Capture Assay

HEK-293FT cells were transfected with pCI/HA-Tau_441_ for 48 h. The cells were lysed in PBS containing protease and phosphatase inhibitors by probe sonication. The debris was removed by centrifugation at 15,000× *g* for 5 min. The cell extract was aliquoted and stored at −80°C.

Various amounts of AD P-tau were dotted on nitrocellulose membrane. After drying for 1 h at 37°C, the membrane was blocked with 5% milk in TBS and incubated with HEK-293FT/HA-tau_441_ cell extract overnight at room temperature. After washing, the captured tau was analyzed by incubating with anti-HA followed by peroxidase-conjugated secondary antibody and developed by using ECL kit as described above.

### Statistical Analysis

The GraphPad Prism 6 software was used for statistical analysis. Results were analyzed by one- or two-way analyses of variance (ANOVA) for multiple-group analysis followed with Tukey’s or Sidak’s multiple comparisons test and by the unpaired or paired two-tailed Student’s *t*-test for two-group comparison.

## Results

### AD P-Tau Induces Tau Aggregation in Tg/hTau Mouse Brains

**Figure 1:**
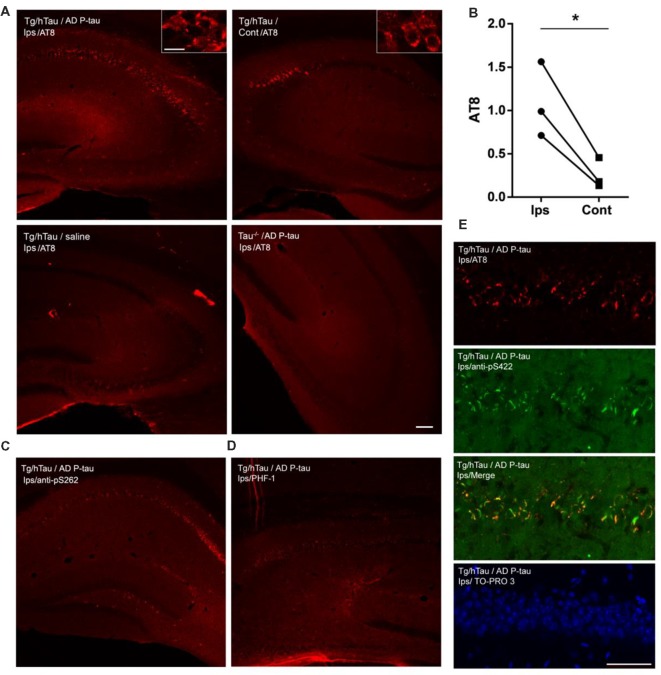
Alzheimer’s hyperphosphorylated and oligomeric tau (AD P-tau)-seeded tau aggregates/pathology in the hippocampus in Tg/hTau mice. **(A–D)** AD P-tau (0.55 μg) or as a control saline was injected unilaterally into the hippocampus of 9–11-month-old Tg/hTau or Tau−/− mice. Coronal brain sections were immunostained with tau antibodies, AT8 (pSer202/Thr205-tau) **(A)**, anti-pSer262-tau **(C)**, and PHF-1 (pSer396/404) **(D)** 10 weeks after injection and immunostaining of ipsilateral (Ips) and contralateral (Cont) hippocampi of Tg/hTau or Tau−/− mice were captured. AT8 staining in 3–4 sections of both ipsilateral and contralateral hippocampi was quantified with ImageJ software from each of three Tg/hTau mice injected with AD P-tau and statistically analyzed with paired student *t*-test **(B)**, *p < 0.05. **(E)** CA1 region of Tg/hTau mouse ipsilateral hippocampus double immunostained with AT8 (Red) and anti-pSer422-tau (Green) and counterstained with TO-PRO 3 iodide for nucleus. Scale bar 100 μm and insert scale bar 20 μm.

We previously found that injection of 0.12 μg AD P-tau into the hippocampus in 3-month-old Tg/hTau mouse induces robust tau pathology 9 months after injection (Hu et al., 2016). In the present study, we injected 0.55 μg AD P-tau unilaterally into the hippocampus of 9-11-month-old Tg/hTau mice, in which no detectable tau pathology occurs at this age (Hu et al., 2016). Similar age tau knockout (Tau−/−) mice were used as a control. Coronal brain sections were immunostained with site-specific and phosphorylation dependent anti-tau antibodies 10 weeks post AD P-tau injection. Robust tau aggregates/pathology was observed in both ipsilateral and contralateral (Figure 1A) hippocampi immunostained with tau antibodies, AT8 (pSer202/Thr205; Figure 1A), anti-pS262-tau (Figure 1C) and PHF-1 (pSer396/404; Figure 1D). No tau pathology was detected in the hippocampus in Tg/hTau mice injected with vehicle or in Tau−/− mice injected with AD P-tau (Figure 1A). AD P-tau seeded tau pathology in the contralateral hippocampus was milder than that in the ipsilateral hippocampus of Tg/hTau mice (Figure 1B). Tau aggregates were co-labeled by AT8 and anti-pS422-tau (Figure 1E). Thus, AD P-tau was able to induce tau aggregation and pathology in Tg/hTau mouse brain 10 weeks post AD P-tau injection, and the tau aggregates were phosphorylated at multiple sites including Ser202/205, Ser262, Ser396/404, and Ser422.

### AD P-Tau Induces Site-Specific Hyperphosphorylation of Tau in Tg/hTau Mouse Brains

**Figure 2:**
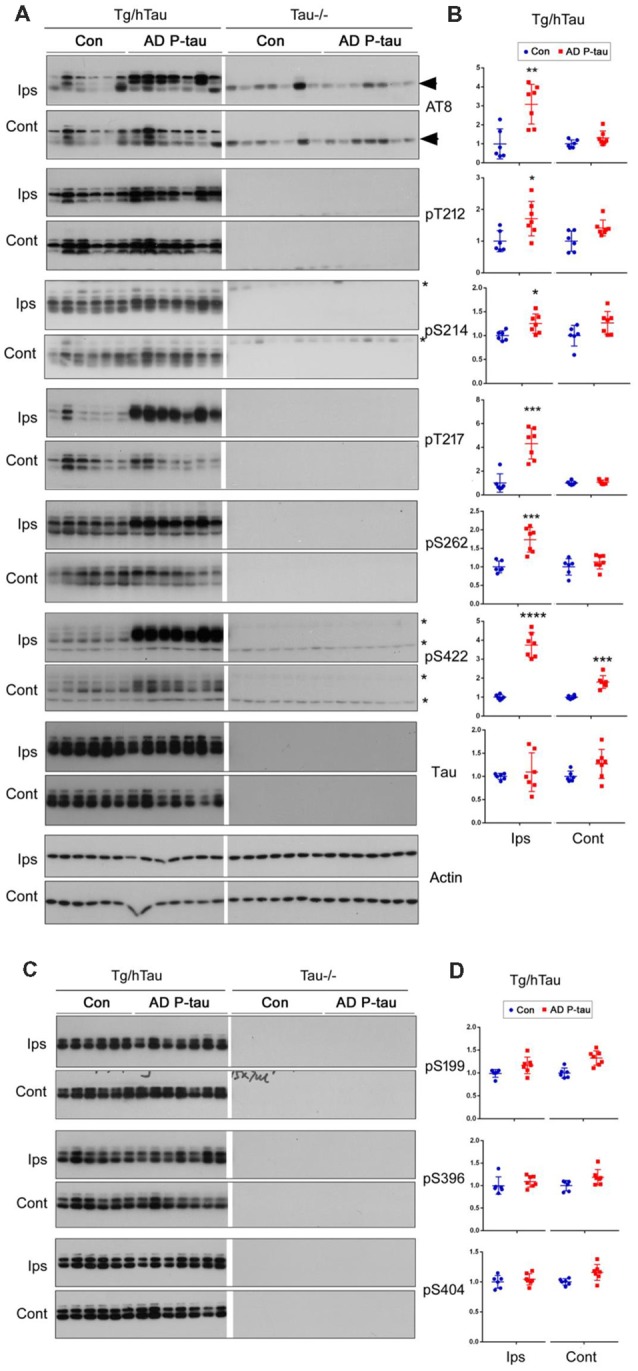
AD P-tau-induced site-specific hyperphosphorylation of tau in the hippocampus in Tg/hTau mice. AD P-tau (0.55 μg) was injected unilaterally into the hippocampus in 9–11-month-old Tg/hTau and Tau−/− mice. Ten weeks post injection, phosphorylation of tau in the hippocampus was analyzed by Western blots developed with the indicated phosphorylation-dependent and site-specific tau antibodies **(A,C)**, normalized with total tau and analyzed with unpaired student *t*-test, and are presented as scattered dots with mean ± SD **(B,D)**, and analyzed with unpaired student *t*-test; *p < 0.05; **p < 0.01; ***p < 0.001; ****p < 0.0001. Arrow head of AT8 blots indicates the heavy chain of IgG (50 kDa) and *indicates the non-specific band.

NFTs are made up of abnormally hyperphosphorylated tau (Grundke-Iqbal et al., 1986a,b). AD P-tau-seeded tau pathology was phosphorylated at multiple sites (Figure 1). To determine whether AD P-tau induces tau hyperphosphorylation, we analyzed the hippocampi of Tg/hTau and Tau−/− mice 10 weeks post AD P-tau injection by Western blots developed with site specific- and phosphorylation-dependent tau antibodies. Tau protein level was found to be similar in the hippocampus in Tg/hTau mice injected with AD P-tau and that in vehicle injected mice (Figures 2A,B). However, phosphorylation of tau was markedly increased at Ser202/Thr205 (AT8), Thr217, Ser262 and Ser422 (Figures 2A,B), was slightly increased at Thr212 and Ser214 (Figures 2A,B), and was not altered at Ser199, Ser396 and Ser404 (Figures 2C,D) in the AD P-tau injected hippocampus in Tg/hTau mice. These results suggest that AD P-tau induces site-specific hyperphosphorylation of tau *in vivo*.

In the contralateral hippocampus, tau phosphorylation was increased only at Ser422 and showed a trend to increase at Thr212 and Ser214 (Figures 2A,B). Ser422 phosphorylation may be an early event in AD P-tau-induced tau hyperphosphorylation. No tau was detected in tau−/− mouse hippocampus (Figure 2A), except a ~50 kDa IgG heavy chain and non-specific bands in the blots developed with AT8 and anti-Ser422, respectively (Figure 2A), confirming immuno-specificity of tau and phosphorylated tau antibodies.

**Figure 3:**
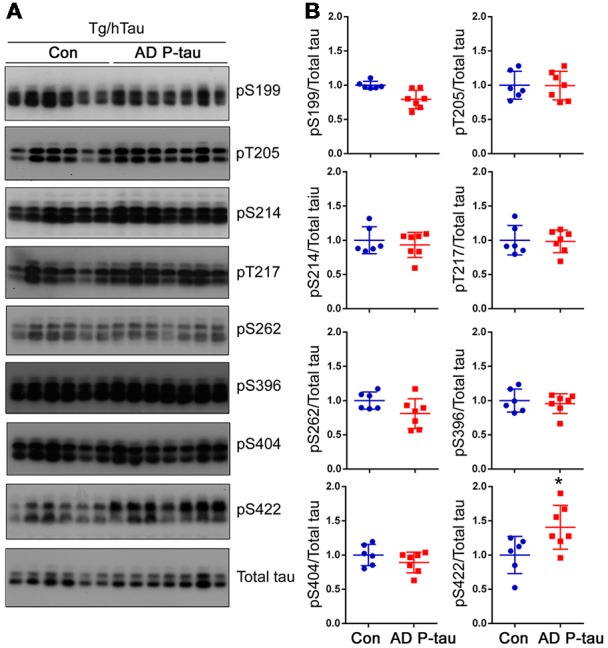
Tau phosphorylation in the ipsilateral cortex in Tg/hTau mice injected with AD P-tau. Phosphorylation of tau at Ser199, Thr205, Ser214, Thr217, Ser262, Ser396, Ser404 and Ser422 in the ipsilateral cortex was analyzed by Western blots **(A)** and analyzed with unpaired student *t*-test. Data are presented as scattered dots with mean ± SD **(B)**; *p < 0.05.

Tau pathology templated by misfolded tau seeds can be propagated to other brain regions (Liu et al., 2012; de Calignon et al., 2012). To study the spread of tau pathological alterations in the cortex, we analyzed tau phosphorylation by Western blots. Similar as in the contralateral hippocampus, phosphorylation of tau at Ser422, but not at Ser199, Thr205, Ser214, Thr217, Ser262, Ser396, or Ser404, was significantly increased in the ipsilateral cortex of Tg/hTau mice injected with AD P-tau (Figure 3). No significant alteration of tau phosphorylation was observed in the contralateral cortex (data not shown). These results also support that phosphorylation of tau at Ser422 may be an early event in AD P-tau templated tau pathogenesis.

### AD P-Tau Seeds Tau to Form SDS- and Reducing Agent-Resistant High-Molecular Weight Aggregation

**Figure 4:**
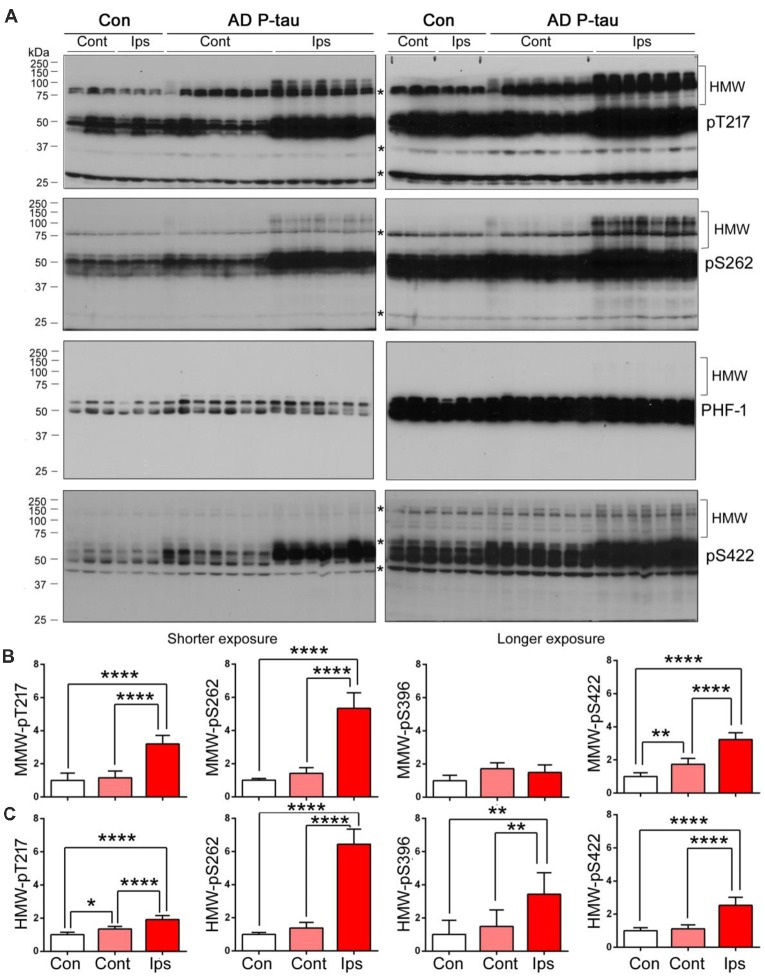
SDS- and β-mercaptoethanol-resistant high molecular weight tau (HMW-tau) in AD P-tau injected hippocampus. Phosphorylation of tau and SDS- and β-mercaptoethanol-resistant HMW-tau in saline or AD P-tau injected hippocampus was analyzed by Western blots developed with anti-pThr217, anti-pSer262, PHF-1, and anti-pSer422 antibodies with shorter (left) and longer (right) exposure to X-ray film **(A)**. Shorter exposure blots were used for quantification of phosphorylation of middle molecular weight tau (MMW-tau), whereas longer exposure blots were used for quantification of SDS- and β-mercaptoethanol-resistant HMW-tau. *Indicates a non-specific band. The data from ipsilateral and contralateral hippocampi in Tg/hTau mice injected with saline were pooled as control since there was no significant difference in tau phosphorylation between them. Levels of phosphorylated MMW-tau **(B)** and HMW-tau **(C)** werestatistically analyzed with one-way ANOVA post Tukey’s multiple comparisons test andare presented as mean ± SD. *p < 0.05; **p < 0.01; ****p < 0.0001.

High molecular tau smear (HMW-tau) in Western blots is only seen in AD brains but not in control human brains, indicating that SDS- and reducing agent-resistant HMW-tau aggregation may be the features of pathological alteration of tau (Zhou et al., 2018). To determine whether AD P-tau is able to induce the formation of HMW-tau in Western blots, we exposed above blots to X-ray film for extended time. We found obvious HMW-tau smears in the blots developed with anti-pT217-tau, anti-pS262-tau and anti-pS422-tau in the ipsilateral hippocampus and no or much less HMW-tau in contralateral hippocampus (Figures 4A,C). Interestingly, we also observed a trace amount of HMW-tau in the ipsilateral hippocampus in the PHF-1 blot (Figures 4A,C). Consistently, phosphorylation of tau at Thr217 and Ser262 was increased only in ipsilateral hippocampus, but at Ser422 was increased in both ipsilateral and contralateral hippocampi (Figures 4A,B). However, compared to saline-injected and contralateral hippocampi, tau phosphorylation at Thr217, Ser262, and Ser422, but not at Ser396/404 (PHF-1), was increased in the ipsilateral hippocampus (Figures 4A,B). Thus, in addition to site-specific hyperphosphorylation, *in vivo* treatment with AD P-tau induces site-specific formation of AD-like SDS- and β-mercaptoethanol-resistant and site-specifically hyperphosphorylated HMW-tau.

**Figure 5:**
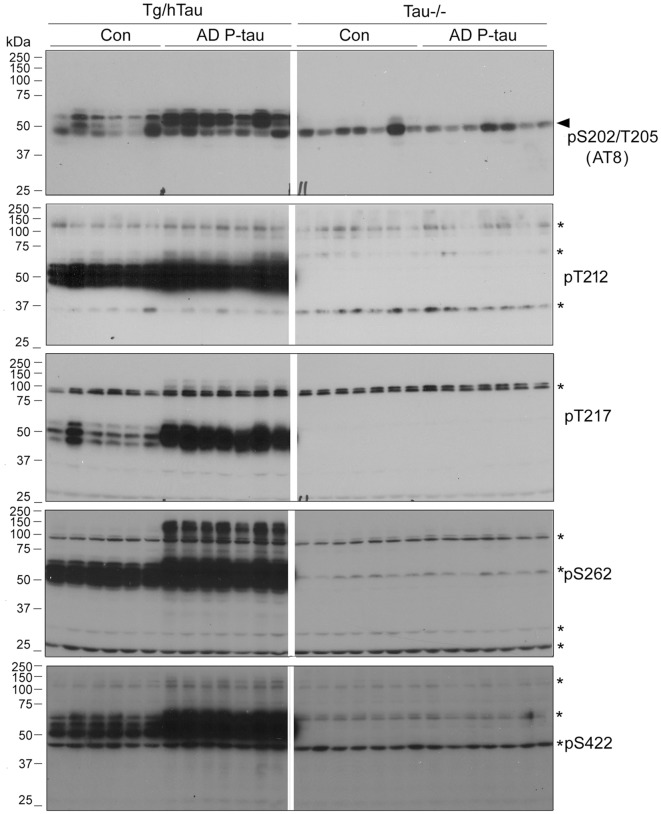
SDS- and β-mercaptoethanol-resistant HMW-tau in AD P-tau injected hippocampus of Tg/hTau mice. Ipsilateral hippocampi from Tg/hTau and Tau−/− mice injected with AD P-tau were analyzed by Western blots developed with AT8 (anti-pS202/T205-tau), anti-pT212-tau,anti-pT217-tau, anti-pS262-tau, and anti-pS422-tau to study site-specific SDS- and β-mercaptoethanol-resistant HMW-tau. *Points the non-specific bands and arrow head indicates the heavy chain of IgG.

To learn that SDS- and β-mercaptoethanol-resistant AD-like HMW-tau in the AD P-tau-injected hippocampus is not the injected exogenous AD P-tau, we analyzed ipsilateral hippocampus of Tg/hTau and Tau−/− mice injected with AD P-tau by Western blots developed with various anti-phospho-tau antibodies. There are obvious HMW-tau seen in anti-pT212, anti-pT217, anti-pS262, and anti-pS422 blots in AD P-tau injected hippocampus of Tg/hTau mice (Figure 5). However, no SDS- and β-mercaptoethanol-resistant HMW-tau in any blots developed with above antibodies was detected in ipsilateral hippocampus of Tau−/− mice injected with AD P-tau (Figure 5). Thus, these data indicate that HMW-tau in Western blots in AD P-tau injected hippocampus is not exogenous protein and it is endogenous tau aggregates induced by AD P-tau.

### Expression of Tau Phosphatases and Kinases in AD P-Tau Injected Hippocampus

**Figure 6:**
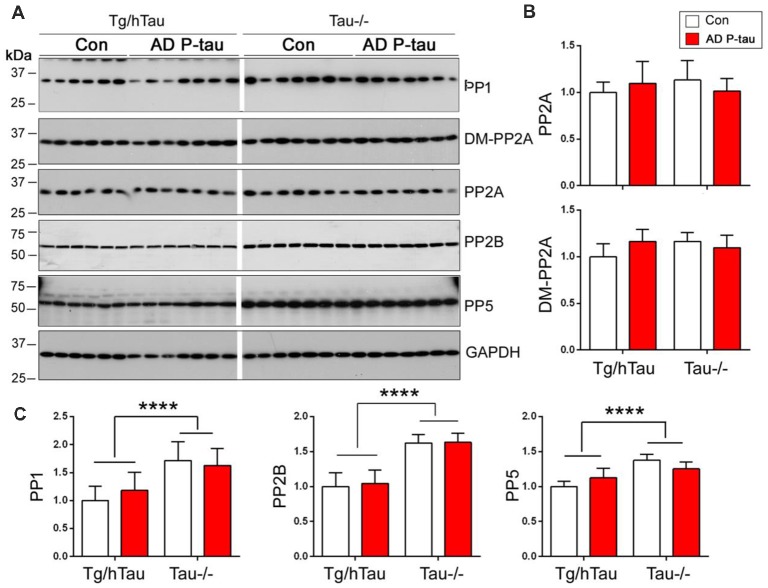
Expression of tau phosphatases in AD P-tau-injected hippocampus. PP1, protein phosphatase 2A (PP2A), demethylated PP2A, PP2B and PP5 in the ipsilateral hippocampus in Tg/hTau and Tau−/− mice injected with AD P-tau or saline were analyzed by Western blots developed with antibodies indicated **(A)**. The levels of demethylated PP2A and PP2A **(B)** and PP1, PP2B and PP5 **(C)** were statistically analyzed with two-way ANOVA post Sidak’s multiple comparisons test after normalized with PP2A (for DM-PP2A) or GAPDH (for PPs) and are presented as mean ± SD. ****p < 0.0001.

PP2A is the major tau phosphatase (Liu et al., 2005). Methylation of PP2A catalytic subunit is required for it to dephosphorylate tau (Sontag et al., 1999, 2004). To learn whether PP2A is involved in AD P-tau-induced site-specific hyperphosphorylation of tau, we analyzed PP2A catalytic subunit and its methylation by Western blots in the hippocampus. We found that levels of PP2A and demethylated PP2A were similar in AD P-tau injected hippocampus as compared with vehicle treatment in Tg/hTau and Tau−/− mice (Figures 6A,B), suggesting that PP2A may not be involved in AD P-tau-induced hyperphosphorylation of tau.

To determine the roles of other tau phosphatases in AD P-tau-induced tau hyperphosphorylation, we analyzed the expression of PP1, PP2B and PP5 by Western blots. Compared with saline injected mice, we did not find any changes of PP1, PP2B or PP5 in the AD P-tau injected hippocampus in Tg/hTau and Tau−/− mice (Figures 6A,C), suggesting that none of these phosphatases are responsible for the hyperphosphorylation of tau. Interestingly, we found that the levels of PP1, PP2B, and PP5 were higher in Tau−/− mouse hippocampus than that in Tg/hTau mouse hippocampus (Figures 6A,C).

**Figure 7:**
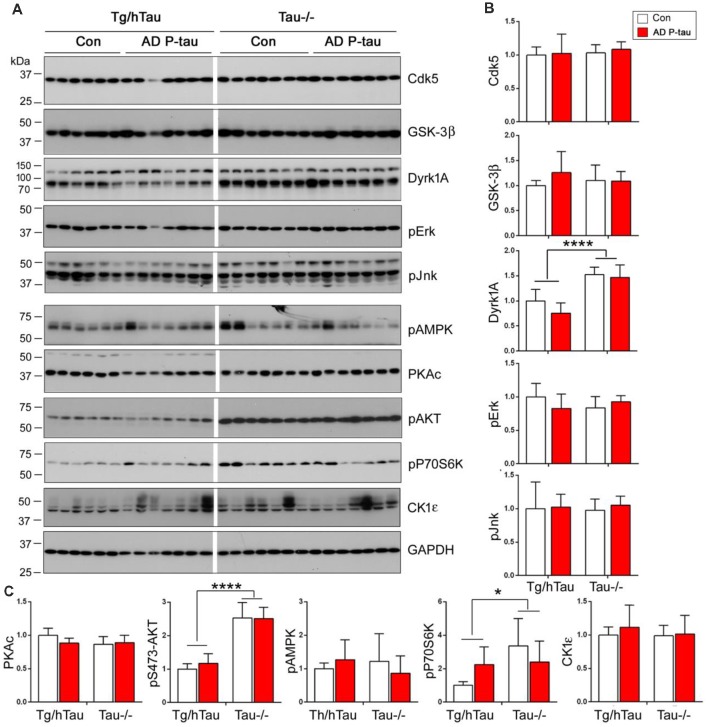
Expression of tau kinases in AD P-tau-injected hippocampi in Tg/hTau and Tau−/− mice. Cdk5, inactive form of GSK-3β (phospho-GSK-3β), Dyrk1A, active form of Erk (phospho-Erk), active form of Jnk/SAPK (phospho-Jnk/SAPK), active form of AMPK (phospho-AMPK), PKA catalytic subunit, active form of AKT (phosphorylated at Ser473), (phospho-AKT), active form of P70S6k (phospho-P70S6K), and CK1ε in ipsilateral hippocampus of Tg/htau and Tau−/− mice injected with AD P-tau or saline were analyzed by Western blots **(A)**. The levels of proline-directed protein kinases (PDPKs; **B)** and non-PDPKs **(C)** were statistically analyzed with two-way ANOVA post Sidak’s multiple comparisons test and are presented as mean ± SD. *p < 0.05; ****p < 0.0001.

AD P-tau-induced hyperphosphorylation of tau at Ser202/Thr205 (AT8), Thr212, Ser214, Thr217, Ser262, and Ser422 (Figures 2A,B), suggesting that both proline-directed protein kinases (PDPKs) and non-PDPKs may participate in the hyperphosphorylation of tau in the AD P-tau injected mouse brains. We analyzed the levels of tau kinases by Western blots. We found that in the AD P-tau-injected hippocampi of Tg/htau and Tau−/− mice, levels of PDPKs, Cdk5, inactive form of GSK-3β (phosphorylated GSK-3β at Ser9), Dyrk1A, active form of Erk (phosphorylated Erk at Thr202/Tyr204), and active form of Jnk/SAPK (phosphorylated Jnk/SAPK at Thr183/Tyr185), were not altered as compared with that in the saline-injected hippocampus (Figures 7A,B), suggesting that they may not contribute to AD P-tau-induced tau hyperphosphorylation. Furthermore, the active form of AMPK (phosphorylated AMPK at Thr172), PKA catalytic subunit, active form of AKT (phosphorylated at Ser473), active form of P70S6K (phosphorylated P70S6K at Thr389), and CK1ε were also not changed in AD P-tau-injected hippocampus as compared with those in corresponding controls (Figure 7C), indicating that these non-PDPKs may not be responsible for the hyperphosphorylation of tau too. Compared with that in the Tg/hTau mouse hippocampus, we found that levels of Dyrk1A, phosphorylated AKT, and phosphorylated P70S6K were increased in the Tau−/− mouse hippocampus (Figures 7A–C).

### Effective Capture of Hyperphosphorylated Tau by AD P-Tau

**Figure 8:**
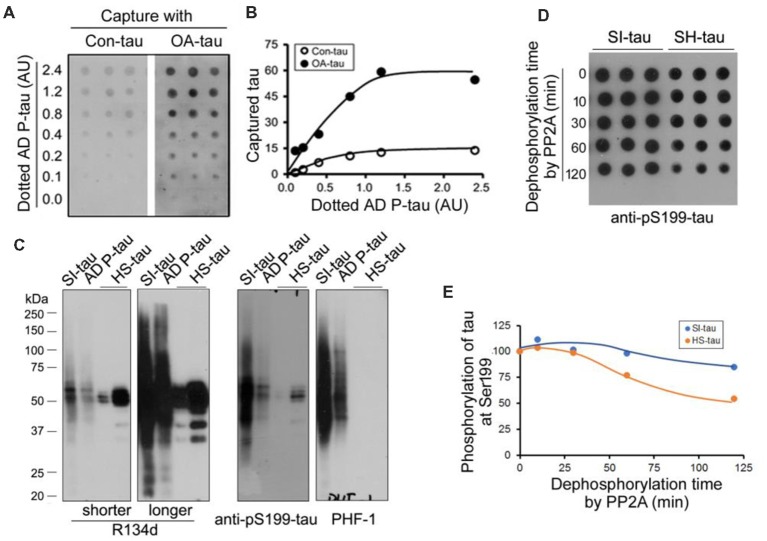
Effects of hyperphosphorylation of tau on its capture by AD P-tau and aggregation of tau on its dephosphorylation by PP2A. **(A,B)** Effect of hyperphosphorylation of tau on its capture by AD P-tau. Tau_441_ tagged with HA was overexpressed in HEK-293FT cells for 48 h. The cells were treated with 100 nM okadaic acid (OA) for 2 h to induce hyperphosphorylation of tau and lysed in PBS containing a cocktail of proteinase and phosphatase inhibitors by probe sonication. AD P-tau was dotted on nitrocellulose membrane and incubated with the 15,000× *g* extract from OA treated cells (OA-tau) or control treated cells (Con-tau) overnight. AD P-tau captured HA-tau_441_ was analyzed by anti-HA, followed by HRP-anti-mouse IgG and ECL **(A)** and plotted against various amount of AD P-tau **(B)**. **(C,D)** Effect of aggregation of tau on its dephosphorylation by PP2A. Sarkosyl insoluble tau (SI-tau), AD P-tau, and heat stable monomeric tau (HS-tau) were isolated from AD cerebral cortex and analyzed by Western blots developed with R134d (pan-tau), anti-pS199-tau and PHF-1 **(C)**. SI-tau and HS-tau were incubated with PP2A (20 mU/ml) for various time points. The phosphorylation of tau at Ser199 was analyzed by dot-blots **(D)** and plotted against time points of dephosphorylation reaction **(E)**.

Above studies suggest no significant alteration of tau phosphatases and tau kinases in the AD P-tau injected hippocampus. To study the possible mechanisms by which AD P-tau induces site-specific hyperphosphorylation of tau *in vivo*, we first assumed that AD P-tau may template phosphorylated tau more effectively. We used overlay capture assay to determine the effect of tau phosphorylation on its capture by AD P-tau, as described previously (Alonso et al., 1996). We overexpressed tau_441_ tagged with HA in HEK-293FT cells and treated the cells with 100 nM okadaic acid (OA) for 2 h to induce tau hyperphosphorylation (Qian et al., 2010). Then, we dotted various amounts of AD P-tau on nitrocellulose membrane and incubated the membrane with the cell-extract of OA-treated (OA-tau) and control-treated (Con-tau) HEK-293FT/tau_441_. AD P-tau captured tau was analyzed by anti-HA and ECL. We found that much more tau was captured by AD P-tau from the extract of OA-tau cells than that from control treated cells (Figures 8A,B), suggesting that hyperphosphorylated tau is captured more effectively by AD P-tau.

### Difficult Dephosphorylation of Aggregated Tau by PP2A

It is well known that aggregated tau is sarkosyl insoluble and heat treatment removes aggregated tau (Greenberg and Davies, 1990; Planel et al., 2007). To learn whether aggregated tau resists to dephosphorylation, we isolated sarkosyl insoluble tau (SI-tau), AD P-tau and heat stable tau (HS-tau) from AD brain and analyzed them by Western blots. These three forms of tau from AD brain showed different patterns in Western blots developed with R134d (pan-tau), anti-pS199-tau and PHF-1 (Figure 8C). SDS- and β-mercaptoethanol-resistant HMW-tau aggregates were present in SI-tau and AD P-tau, but not in HS-tau (Figure 8A), confirming aggregated tau in SI-tau and AD P-tau and the non-aggregated nature of HS-tau (Grundke-Iqbal et al., 1986a; Greenberg and Davies, 1990). AD P-tau was used for above capture assay. SI-tau and HS-tau were used as aggregated tau and non-aggregated tau to study efficiency of dephosphorylation by PP2A. We dephosphorylated SI-tau and HS-tau with PP2A for various time points and analyzed dephosphorylation efficiency by dot blots developed with anti-pS199-tau since HS-tau was phosphorylated at Ser199. Even PP2A could not dephosphorylate tau at Ser199 effectively (Liu et al., 2005), we found that Ser199 phosphorylation of SI-tau and of HS-tau was decreased in time dependent manner by PP2A (Figures 8D,E), and HS-tau was dephosphorylated more rapidly than SI-tau (Figures 8D,E), suggesting PP2A dephosphorylates the non-aggregated tau more effectively than the aggregated tau.

## Discussion

Tau oligomers recently have emerged as the pathogenic species in tauopathies. AD P-tau isolated from AD brain is hyperphosphorylated and oligomeric (Köpke et al., 1993). It serves as potent tau seeds, sequesters/captures normal tau *in vitro* (Alonso et al., 1996) and induces tau pathology *in vivo* (Hu et al., 2016; Dai et al., 2018). In the present study, we unilaterally injected AD P-tau into the hippocampus of 9–11-month-old Tg/hTau mice and found for the first time that in addition to the induction of tau aggregation/pathology, AD P-tau led to site-specific hyperphosphorylation and SDS- and reducing agent-resistant and AD-like high-molecular weight tau *in vivo* 10 weeks post injection. Tau aggregation/pathology was observed in both hippocampi, but four-times less in contralateral sites. In the AD P-tau injected hippocampus, tau was abnormally hyperphosphorylated at Ser202/Thr205, Thr212, Ser214, Thr217, Ser262, and Ser422. The SDS- and reducing agent-resistant and AD-like HMW-tau was hyperphosphorylated at Ser212, Ser217, Ser262, and Ser422. Different from ipsilateral hippocampus, tau was hyperphosphorylated at Ser422 in the contralateral hippocampus and the ipsilateral cortex. No detectable alteration in levels of tau phosphatases and kinases was observed in AD P-tau injected hippocampus. However, we found that hyperphosphorylated tau was more effectively captured by AD P-tau and aggregated tau was relatively resistant to dephosphorylation by PP2A. Thus, we speculate that AD P-tau seeds site-specifically hyperphosphorylated tau to form aggregates, and the aggregated tau resists to dephosphorylation by PP2A, leading to hyperphosphorylation and pathology of tau.

Abnormally hyperphosphorylated tau is the major component of NFTs (Grundke-Iqbal et al., 1986b). Tau aggregates induced by misfolded tau seeds are labeled by various site-specific and phosphorylation-dependent antibodies (Clavaguera et al., 2009; Hu et al., 2016), but the state of tau phosphorylation in misfolded tau seeds-injected brains had not been documented biochemically. In the present study, we found that tau phosphorylation was increased at Ser202/Thr205, Thr212, Ser214, Thr217, Ser262, and Ser422, but not at Ser199, Ser396 and Ser404, in the AD P-tau-injected hippocampus, suggesting that AD P-tau induces site-specific hyperphosphorylation of tau *in vivo*. Since these hyperphosphorylated sites are followed by both proline and non-proline residues, both PDPK or non-PDPK may be involved in AD P-tau-induced hyperphosphorylation of tau. The major PDPK of tau is GSK-3β, which phosphorylates tau at Ser199, Ser202, Thr205, Thr212, Thr217, Ser396, and Ser404 with Ser199, Thr205 and Ser396 being the most favorable sites in cells (Liu et al., 2006; Qian et al., 2010). However, Ser199 and Ser396 phosphorylation was not increased, suggesting that GSK-3β may not be involved in the hyperphosphorylation induced by AD P-tau *in vivo*. Moreover, we did not find a significant alteration in phospho-Ser9 of GSK-3β in AD P-tau injected hippocampus, supporting that GSK-3β probably does not contribute to AD P-tau-induced tau hyperphosphorylation. In case of PKA, it phosphorylates Ser214 more effectively than Ser262, but more increase of tau phosphorylation at Ser262 than Ser214 was seen in the AD P-tau-injected hippocampus. Similarly, other kinases, including Cdk5, Erk1, Jnk/SAPK, Dyrk1A, P70S6K, AMPK, AKT, and CK1ε, may not be involved in AD P-tau-induced hyperphosphorylation was evidenced by the site-specific phosphorylation and their expression levels. Interestingly, we found that levels of PP1, PP2B, PP5, phospho-AKT, Dyrk1A and phospho-P70S6k were increased in tau−/− hippocampus, suggesting that tau may influence the expression or/and degradation of these proteins directly or indirectly, which remain to be studied.

PP2A is the major tau phosphatase (Liu et al., 2005) and it dephosphorylates multiple sites of tau with different efficiencies (Liu et al., 2005). PP2A also dephosphorylates GSK-3β, resulting in its activation (Qian et al., 2010; Wang et al., 2015a). Inhibition of PP2A increases tau phosphorylation directly and indirectly through activating GSK-3β (Qian et al., 2010). Methylation of PP2A catalytic subunit at Lys 309 enhances its activity to dephosphorylate tau (Sontag et al., 1999, 2004). However, no alteration of methylated PP2Ac or Ser9 phosphorylation of GSK-3β was observed in AD P-tau injected hippocampus, suggesting PP2A may not be course of hyperphosphorylation of tau in AD P-tau-injected mouse brain. Similarly, similar levels of PP1, PP2B and PP5 in AD P-tau-injected and saline-injected hippocampi suggest that they may not play roles in AD P-tau-induced tau phosphorylation.

Among these hyperphosphorylation sites induced by AD P-tau *in vivo*, phosphorylation of tau at Ser422 was very interesting. Increase of Ser422 phosphorylation was found in the contralateral hippocampus and the ipsilateral cortex, where very limited tau pathology were observed. Thus, Ser422 phosphorylation may be an early event in AD P-tau-induced tau pathology. Several kinases are able to phosphorylate Ser422, including Jnk/SAPK (Wang and Liu, 2008). PP2A and PP5 effectively dephosphorylate Ser422 (Liu et al., 2002). Treatment of brain slices with OA induces tau hyperphosphorylation at multiple sites, with the most increase at Ser422 (Gong et al., 2000; Qian et al., 2010). Ser422 is abnormally hyperphosphorylated in AD brain (Liu et al., 2009). Normal tau protein has been proposed to have a “paper clip” structure, in which the N- and C-terminal ends fold over the microtubule-binding domain to prevent the protein from self-aggregation (Mandelkow et al., 2007). Ser422 phosphorylation may make tau C-terminus to stretch out and expose the microtubule-binding domain, thereby leading to tau captured easily by AD P-tau. Specially, Ser422 was the only site to be hyperphosphorylated in the ipsilateral and contralateral hippocampi and the ipsilateral cortex of AD P-tau injected Tg/hTau mice. Thus, Ser422 phosphorylation may be critical in AD P-tau-induced aggregation/tau pathology, but this remains to be determined in future studies.

AD P-tau sequesters tau *in vitro* and induces tau aggregation *in vivo* (Alonso et al., 1996; Hu et al., 2016). We found that hyperphosphorylated tau induced by OA in cells was captured by AD P-tau more effectively than normal tau from control treated cells. OA is a PP2A inhibitor (Bialojan et al., 1988). Treatment of brain slices with OA induces tau hyperphosphorylation at multiple sites, including Ser422 and Ser262 phosphorylation (Gong et al., 2000; Qian et al., 2010). Tau in NFTs is hyperphosphorylated (Grundke-Iqbal et al., 1986b). In the present study, we found that PP2A dephosphorylated heat-stable monomeric tau more effectively than aggregated sarkosyl-insoluble tau. We previously reported that tau is rapidly dephosphorylated during postmortem delay (Wang et al., 2015b). In AD brain, SI-tau was hyperphosphorylated at all the sites studied, but HS-tau was phosphorylated only at Ser199, which also supports that aggregated tau may resist to dephosphorylation during postmortem period. Dephosphorylation with alkaline phosphatase abolishes the ability of AD P-tau to aggregate with normal tau and prevents tangle formation (Alonso et al., 1996). From these findings, we speculate that AD P-tau captures phosphorylated tau and that aggregated tau is resistant to dephosphorylation, leading to tau hyperphosphorylation. Hyperphosphorylated tau aggregates capture and template tau aggregation and eventually leading to tau pathology.

## Author Contributions

JM, RS, LL, FC, YZ, YT and WH performed experiments and analyzed the results. C-XG and KI provided the reagents, discussed results and edited the manuscript. FL designed and performed experiments, analyzed and interpreted results, and wrote the manuscript.

## Conflict of Interest Statement

The authors declare that the research was conducted in the absence of any commercial or financial relationships that could be construed as a potential conflict of interest.
